# Evolution of Pre- and Post-Copulatory Traits in Male *Drosophila melanogaster* as a Correlated Response to Selection for Resistance to Cold Stress

**DOI:** 10.1371/journal.pone.0153629

**Published:** 2016-04-19

**Authors:** Karan Singh, Manas Arun Samant, Megha Treesa Tom, Nagaraj Guru Prasad

**Affiliations:** Indian Institute of Science Education and Research Mohali, Knowledge City, Sector 81, SAS Nagar, PO Manauli, Punjab, 140306, India; National Cancer Institute, UNITED STATES

## Abstract

**Background:**

In *Drosophila melanogaster* the fitness of males depends on a broad array of reproductive traits classified as pre- and post-copulatory traits. Exposure to cold stress, can reduce sperm number, male mating ability and courtship behavior. Therefore, it is expected that the adaptation to cold stress will involve changes in pre- and post-copulatory traits. Such evolution of reproductive traits in response to cold stress is not well studied.

**Methods:**

We selected replicate populations of *D*. *melanogaster* for resistance to cold shock. Over 37–46 generations of selection, we investigated pre- and post-copulatory traits such as mating latency, copulation duration, mating frequency, male fertility, fitness (progeny production) and sperm competitive ability in male flies subjected to cold shock and those not subjected to cold shock.

**Results:**

We found that post cold shock, the males from the selected populations had a significantly lower mating latency along with, higher mating frequency, fertility, sperm competitive ability and number of progeny relative to the control populations.

**Conclusion:**

While most studies of experimental evolution of cold stress resistance have documented the evolution of survivorship in response to selection, our study clearly shows that adaptation to cold stress involves rapid changes in the pre- and post-copulatory traits. Additionally, improved performances under stressful conditions need not necessarily trade-off with performance under benign conditions.

## Introduction

Reproductive traits of insects are sensitive to thermal stress [1–11]. In promiscuous species like *D*. *melanogaster*, male reproductive success is dependent on a large set of traits that can be broadly classified into pre- and post-copulatory traits. In order to successfully mate, the male has to perform a series of intricate courtship behaviors [12, 13]. Additionally, since females in this species store sperm from multiple males, post-copulatory traits which may determine sperm competitive ability are crucial components of male fitness [14]. Male fitness is affected by a number of sperm related traits such as numbers, quality, motility and morphology [15, 16]. The seminal fluid contains accessory gland proteins (Acps) that can also influence female behaviour and thereby affect sperm competition [17–20]. It is well established that both sperm and Acps influence post-copulatory sexual selection in insects [18, 21]. Environmental stress can majorly affect the quality of ejaculates and can therefore affect male fitness [22–24]. Multiple previous studies have shown that temperature stress affects the pre- and post-copulatory traits of male insects. Temperature shock can adversely affect several pre-copulatory traits such as male fertility [8, 25–28], courtship behaviour, mating success [10, 29, 30], copulation duration [9] and mating latency [2, 27, 31, 32, 33–35].

Specifically, cold shock is also known to affect male post-copulatory traits. When *D*. *melanogaster* males are subjected to cold shock, sperm in the seminal vesicle are known to become immobile [36]. Motile sperm are not detected in the vesicles for over twenty four hours post cold shock. The females need to eject these immobile sperm and mate again to ensure future fitness [36]. Given the effects of temperature on the male reproductive traits, it is expected that adaptation to cold shock will involve changes in pre- and post-copulatory traits.

Hence, in the present study, our primary aim is to investigate the evolution of the pre-and post-copulatory traits in the populations of *D*. *melanogaster* selected for increased resistance to cold shock [37]. We investigated the pre- and post-copulatory traits i.e., mating latency (time required to start mating), copulation duration (the duration for which the mating pair remains in copula), mating success, male fertility, progeny production and sperm competitive ability with and without cold shock in populations of *D*. *melanogaster* selected for increased resistance to cold shock and their controls. These studies were carried out between 37–46 generations of selection.

Our findings indicate rapid evolution of sperm competition and other reproductive traits (i.e., mating latency, mating success, male fertility and progeny production) post cold shock in our selection populations.

## Materials and Methods

The derivation and maintenance of all populations of *D*. *melanogaster* used for this study has been detailed in Singh et al. [37]. A brief summary is presented below.

### Derivation and maintenance of selected (FSB 1–5) and control (FCB1-5) populations

The selected and the control populations were derived from five replicate BRB ancestral populations (BRB 1–5). The BRB populations were established in 2011 by combining equal numbers of males and females from each of 19 isofemale lines. The isofemale lines were themselves established using wild-caught females from Blue Ridge Mountains, Georgia, USA. These isofemale lines were initially maintained in the laboratory of Prof. Daniel Promislow and were provided to us as a gift in 2010. The BRB populations were maintained under a standard laboratory condition (25°C temperature, 50–60% relative humidity, and 12 hours-12 hours light/dark cycle) for 35 generations before the start of the current study. Each BRB population was maintained on a 14 day discrete generation cycle at the standard laboratory condition as mentioned above with an effective population size equivalent to 2800 individuals. From each of the BRB 1–5 populations, one selected (FSB) and one control (FCB) population were derived. For example, FSB 1 and FCB 1 were derived from BRB 1, FSB 2 and FCB 2 from BRB 2 and so on. FSB 1–5 and FCB 1–5 populations were maintained on a 13 day discrete generation cycle at a standard laboratory condition as mentioned above. These FSB 1–5 and FCB 1–5 populations were maintained in the following manner. On the 12^th^ day post egg collection, the adult flies were transferred to empty glass vials (25 mm diameter × 90 mm height) and a cotton plug was pushed in such that the flies were restricted to the bottom one-third of the vial. Vials containing flies from FSB population were subjected to a temperature of -5°C for one hour in ice-salt-water slurry. Immediately after the cold shock were transferred to Plexiglas cages and provided Petri-plates containing banana-yeast-jaggery food. Twenty hours post cold shock fresh food plates were provided for duration of 18 hours. In order to start the next generation, eggs were collected from these food plates and transferred to food vials at a density of ~100 eggs per vial containing 6 ml of banana-yeast-jaggery food. Twenty such vials were set up for each of the five FSB populations. As egg viability post cold shock is ~70% of the 100 eggs collected into each vial about 70 individuals made it to adulthood. FCB populations were maintained in conditions identical to the FSB populations, except that the vials containing flies from the FCB populations were exposed to a temperature of 25°C in water-bath for one hour. To control for larval density, eggs were collected at a density of ~70 eggs/vial. Each FSB and FCB population had close to 1400 adult individuals.

### Standardization of flies

To account for the non-genetic parental effects [38], each of the five FSB populations (FSB 1–5) and the five FCB populations (FCB 1–5) were put through one generation of common rearing. This process is referred to as standardization and the flies generated are referred to as standardized flies. To standardize flies, the eggs were collected at a density of 70 eggs/vials from each of FSB1-5 and FCB1-5 populations and were reared at the standard laboratory condition as describe above. Twenty vials were established for each population. On the 12^th^ day post egg collection, for each population, 20 vials of flies (approximately 1400 individuals) were transferred into Plexiglas cages and provided a Petri plate containing banana-yeast-jaggery food. In order to generate experimental flies, eggs were collected at a density of 70 eggs/vial from these cages for each of the five FSB and FCB populations and reared at standard laboratory conditions as mentioned above.

### Experimental protocol

#### Generation of experimental flies

Experiments 1.1, 1.2, 1.3 and Experiment 2 were carried out after 37, 39, 40 and 45 generations of selection respectively. For each of these experiments, following one generation of standardization (see above), eggs were collected at a density of 70 eggs/vial from each of the 10 populations (5 FSB and 5 FCB populations). For each of the FSB and FCB populations 16 vials were set up. The vials were incubated at standard culture conditions (25°C temperature, 50–60% relative humidity, and 12 hours-12 hours light/dark cycle). On the 9–10^th^ day post egg collection virgin male flies were collected at a very young stage (≤ 4 hours post eclosion) using light CO_2_ anaesthesia. It was ensured that the collected males were from the peak of the eclosion distribution. These males were housed in single-sex vials containing 2 ml of banana-yeast-jaggery food at a density of 10 males per vial until the 12^th^ day post egg collection when mating trials were conducted. Thus the males used in the experiments were roughly 2 to 3 days old as adults.

#### Generation of common females from ancestral BRB population

In order to record the pre- and post-copulatory traits of males belonging to the two selection regimes, they were housed with ancestral BRB females. To collect eggs from which these females would be generated, fresh food plates were given to corresponding BRB population for 6 hours. Then from these plates eggs were collected at a density of 70 eggs/vial containing 6 ml of banana-yeast-jaggery food. For each population 28 vials were set up and incubated at standard laboratory conditions as described above. On 9–10^th^ days post egg collection, virgin females were collected using mild CO_2_ anaesthesia as described above. Virgin females were held individually in vials provisioned with 2 ml of food. Vials containing flies were incubated at standard laboratory condition until the start of the mating trial assay.

#### Generation of common females and competitor males carrying a recessive genetic marker

To assess the fertilization success of the experimental males, we used flies from an outbred laboratory population–LH_st_ [39]. This population carries a recessive autosomal eye color marker–scarlet eye–thereby allowing quantification of fertilization success (see below, Experiment 2 in this section). Previous experiments from our lab show that the scarlet eye colour marker has no discernible effect on the behaviour and fitness of flies [40]. LH_st_ flies were grown under similar conditions (70 eggs/vial in 6 ml of banana-yeast-jaggery food, 25°C temperature, 50–60% relative humidity, and 12 hours:12 hours light/dark cycle). LH_st_ males (competitors) and females were collected as virgins, as described above, and held individually in vials for 2–3 days until the mating trials.

#### Cold shock treatments

We have followed the same protocol for cold shock treatments as described in Singh et al. [37] with minor modifications. Briefly, on the 12^th^ day post egg-collection virgin FSB and FCB male flies were transferred to clean and dry glass vials (25 mm diameter × 90 mm height) at a density of 50 individuals per vial. The cotton plug was pushed in such that the flies were confined to a small area at the bottom one third of the vial. Following this, the vials were placed for one hour in ice-salt-water slurry maintained at -5°C. Care was taken so that the part of the vial containing the flies was completely immersed in the ice-salt-water slurry. Immediately, after one hour of the cold shock, the flies were transferred into a Plexiglas cage (14 cm length × 16 cm width × 13 cm height) containing a Petri plate of banana-yeast-jaggery food and maintained at standard laboratory conditions (see above). For each of the populations, one hour before the start of mating trials, experimental flies were aspirated out from the cages and housed singly in vials containing 2 ml of banana-yeast-jaggery food.

#### No shock treatment

The FSB and FCB flies for the control treatment (no shock treatment) were handled in a manner identical to the cold shock treatment except that the vials containing male flies were placed for one hour in a water-bath maintained at 25°C instead of -5°C.

### Experiment 1.1: The effect of cold shock on pre- and post-copulatory traits

Single pair experimental design was used to quantify the effects of selection regime on mating latency, copulation duration, fertility and progeny production post cold shock. This experiment was carried out after allowing cold shocked males from the FSB and FCB populations to recover for different periods of time (4, 12 or 30 hours) post cold shock. Note that we used a different set of flies at each of the three time points. For each of the three time points, a virgin BRB female was combined with one of the cold shocked FSB or FCB males in a vial (25 mm diameter × 90 mm height) provisioned with food. The cotton plug covering the vial was pushed deep into the vial such that the flies were restricted to the bottom one third of the vial. The pair was observed continuously for two hours for mating latency and copulation duration. For male fertility assay, after a single mating was over, the female was immediately separated using mild CO_2_ anesthesia and the male was discarded. The female was transferred into a fresh food vial and allowed to oviposit for 24 hours. Following this, the female was discarded and the vial was held for another 24–30 hours to check egg hatchability. A male was considered fertile if at least one egg hatched. The sample size of the mating trials varied across recovery periods. For the mating trial held after 4 hours of recovery post cold shock, 70 males from each FSB and FCB population were used while those conducted after 12 and 30 hours of recovery used 60 males from each FCB and FSB population.

### Experiment 1.2: Progeny production of ancestral females mated with cold shocked males

Progeny production of ancestral females was assayed using a protocol similar to the one described in the previous experiment (Experiment 1.1), except that, the effect of mating with cold shocked males (FSB or FCB) on progeny production by females was assayed only at two-time points namely, 4 and 12 hours post cold shock to males. Again for each of the two time points, different flies were used. For each time point, after combining one cold shocked male from either FSB or FCB population with one baseline (BRB, not subjected to cold shock) female per vial, vials were kept undisturbed for two hours. Following this, the females from all vials were quickly separated under light CO_2_ anaesthesia and were held individually in test tubes containing banana-yeast-jaggery food to oviposit for 24 hours to measure ‘day one’ progeny production. Twenty hours later, the same females were transferred to new test tubes containing banana-yeast-jaggery food to oviposit for 24 hours in order to measure ‘day two’ progeny production. On the 13^th^ day post oviposition, the progeny emerging from these test tubes were counted, yielding a value of female fitness. Total number of progeny from each of the test tubes was used as the unit of analysis. The data was separated into two groups- (a) The number of offspring produced by the females which yielded at least one viable progeny (b) The number of females which did not produce any progeny (i.e., zero fitness). These data were analysed separately. When a female produced no progeny, it could be because the female did not mate or mating happened but the female did not receive fertile sperm from its mate. Since we did not observe for mating in this experiment, we cannot distinguish between these possibilities.

### Experiment 1.3: Pre- and post-copulatory traits in males not subjected to cold shock

After subjecting 300 virgin males from each of the FSB and FCB populations to the no shock treatment, they were transferred into a Plexiglas cage and provided a fresh banana-yeast-jaggery food plate. Three hours later 35 randomly chosen males from each population were aspirated out and transferred individually to separate vials provisioned with banana-yeast-jaggery food. One hour later each of these males was held with a single virgin female from the BRB population in a food vial. The cotton plug was pushed down into the vial so that the space available to the flies was the bottom one-third of the vial. The vials were observed for mating latency and copulation duration. The vials were observed until a single mating was over. To assess the ability of FCB and FSB males to influence female progeny production, the male and the female from each vial were immediately sorted under light CO_2_-anaesthesia and the male was discarded. Each female was individually transferred into a fresh food vial containing 6 ml of banana-yeast-jaggery food for oviposition for two successive 24 hours periods, following which the female was discarded. Thirteen days later the progeny emerging from these vials were counted. Total number of progeny from each vial was used as the unit of analysis.

### Experiment 2: Effect of selection regime on sperm offense (P2) ability

A virgin LH_st_ female and a LH_st_ male were combined in a vial provisioned with banana-yeast-jaggery food. The cotton plug was pushed deep into the vial to restrict the flies to bottom one third of the vial. The pair was observed for successful mating for a period of one hour. Once a single mating was over, the male and the female were immediately sorted using mild CO_2_-anaesthesia and the male was discarded. The female was transferred back into the vial and allowed to recover from CO_2_-anaesthesia for half an hour. After this, the female was combined with one of the experimental, FSB or FCB males (which had been either cold-shocked 12 hours before or not shocked). Vials were left undisturbed for 24 hours to let the experimental male and the LH_st_ female interact. Following this, the females were transferred individually into banana-yeast-jaggery-food vials to oviposit for 18 hours. After that, the females were discarded. Thirteen days later, the numbers of red-eyed and scarlet-eyed flies were recorded amongst the progeny. As the LHst flies are true breeding scarlet eyed flies (with the scarlet eye colour marker being recessive) while the experimental FSB and FCB flies are wild type (red eyed) flies, any progeny sired by the first male were scarlet eyed and those sired by the second (i.e., experimental) males were red eyed. For the sperm offense ability P2 assay 80 males were used for the “Cold shock” treatment and 50 males for the “No shock” treatment from each of the 10 populations (5 FSB and 5 FCB). To measure P2 from each vial, we used data from only those vials that showed at least one red-eyed progeny (i.e., the second male had non-zero fitness). The proportion of red eyed progeny (P2) was calculated for each vial and was used as the unit of analysis. The final sample size for each population was between 29 and 60. The vials in which females failed to produce even a single red eyed progeny (i.e., second male had zero fitness) were analysed separately. For each population we calculated the proportion of females that did not produce even a single red eyed progeny and used this as a unit of analysis.

### Statistical analysis

Since the selected and the control populations having the same numerical subscript originated from the same BRB ancestral population, they are more closely related to each other than they are to populations with a different numerical subscript. For example, FSB 1 is more closely related to FCB 1 (since they both were derived from BRB 1) than to FSB 2. Hence, populations with the same numerical subscript are treated as statistical blocks in all the analyses. For Experiment 1.1 mating latency, copulation duration, mating success and male fertility were analysed using a three-factor mixed model analysis of variance (ANOVA) with selection regime (FCB vs. FSB) and period (4, 12 and 30 hours post cold shock) as fixed factors crossed with block as a random factor. Multiple comparisons were performed using Tukey’s HSD.

Data from Experiment 1.2, i.e. progeny production of ancestral females mated with cold shocked males, were analysed using four-factor mixed model analysis of variance (ANOVA) with selection regime (FCB vs. FSB), period (4 vs. 12 hours post cold shock) and Day (Day 1 vs. Day 2) as fixed factors crossed with Block (1–5) as random factor. We also analysed the proportion of females which did not produce any progeny. Data on the proportion of females that produced progeny were analysed using a three-factor mixed model ANOVA treating selection regime (FSB and FCB), period (4 and 12 hours of recovery post cold shock) as fixed factors crossed with random blocks (1–5).

Mating latency, copulation duration, mating success, male fertility and progeny production data from Experiment 1.3 were analysed using a two-factor mixed model analysis of variance with selection regime (FCB vs. FSB) crossed with block as a random factor.

For Experiment 2, proportion of red eyed progeny and proportion of females which produced no red eyed progeny were analysed using a three-factor mixed model analysis of variance with selection regime (FCB vs. FSB) and treatment (Cold shock vs. No shock) as fixed factor crossed with blocks as random factor. All the analyses were done using JMP 10 (SAS Institute, Cary, NC, USA).

## Results

### Experiment 1.1: Effect of cold shock on pre- and post-copulatory traits

#### (a) Mating latency

**Fig 1:**
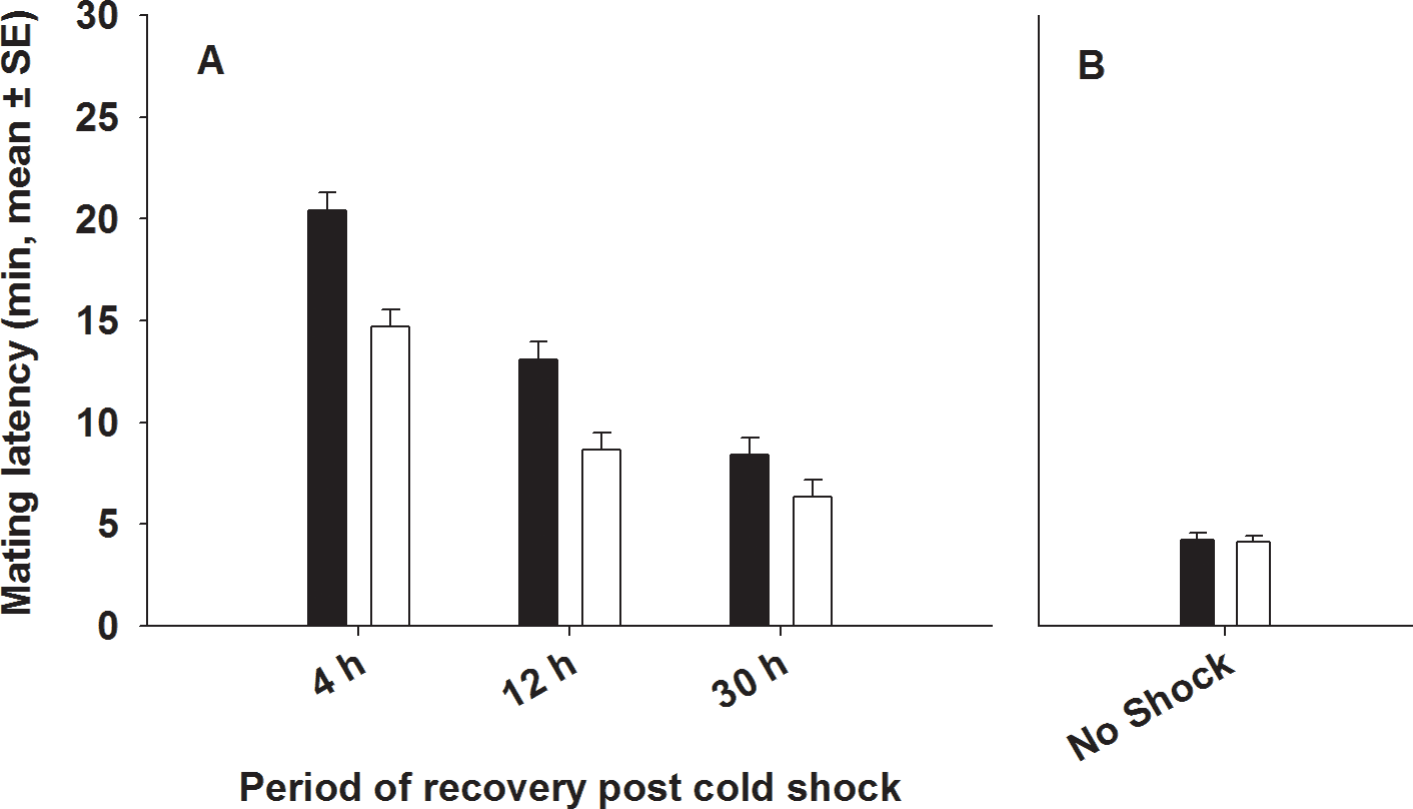
**Effect of cold shock (A) or no shock (B) on mating latency.** (A) We assayed mating latency at 4 hours (h), 12 h and 30 h post cold shock. Closed bars represent FCB and open bars represent FSB populations. Selection and period had significant effects on mating latency. However, selection × period interaction was not significant. (B) Under no-shock treatment, there was no significant difference in mating latency between FSB and FCB males.

Mating latency has evolved in response to selection. We found significant effects of the selection (FSB and FCB), period (4, 12 and 30 hours) and block (1–5) on mating latency (Table 1A, Fig 1A). FSB males on an average start mating 4 minutes earlier than FCB males after being subjected to cold shock (and combined with virgin base line females) (Fig 1A). Multiple comparisons employing Tukey’s HSD indicated that in both FSB and FCB populations, mating latency measured at 4 hours post cold shock was significantly higher relative to mating latency measured at 12 and 30 hours post cold shock. There was no significant selection × period interaction (Table 1A, Fig 1A). While block had a significant effect, none of the interactions involving block were significant (Table 1A).

**Table 1:** Effect of cold shock on pre- and post-copulatory traits. Summary of results of a three-factor mixed model ANOVA considering selection regime (FSB and FCB) and period (recovery period 4, 12 and 30 hours after cold shock) as fixed factors crossed with block as random factor on the (A) mating latency, (B) copulation duration data. *p*-values in bold are statistically significant. SS: Numerator sum of squares, MS Num: Numerator mean square, DF Num: Numerator degrees of freedom, DF Den: Denominator degrees of freedom.

Trait	Effect	SS	MS Num	DF Num	DF Den	*F* ratio	*p*
(A)	Selection (Sel)	5914.456	5914.456	1	4.037	18.547	**0.012**
Mating	Period (Per)	25814.136	12907.068	2	8.052	25.368	**<0.001**
latency	Block (Blk)	11609.018	2902.254	4	6.897	4.440	**0.043**
	Sel×Per	805.357	402.679	2	8.152	2.279	0.164
	Sel×Blk	1277.863	319.466	4	8.017	1.811	0.220
	Per×Blk	4086.438	510.805	8	8	2.897	0.077
	Sel× Per×Blk	1410.611	176.326	8	1449	0.765	0.634
(B)	Selection (Sel)	118.852	118.852	1	4.022	1.520	0.285
Copulation	Period (Per)	278.046	139.023	2	8.041	1.497	0.280
duration	Block (Blk)	766.883	191.721	4	4.175	1.697	0.306
	Sel×Per	20.966	10.483	2	8.065	0.179	0.839
	Sel×Blk	313.955	78.489	4	8.007	1.336	0.336
	Per×Blk	746.312	93.289	8	8	1.587	0.264
	Sel× Per×Blk	470.204	58.775	8	1449	1.776	0.077

#### (b) Copulation duration

**Fig 2:**
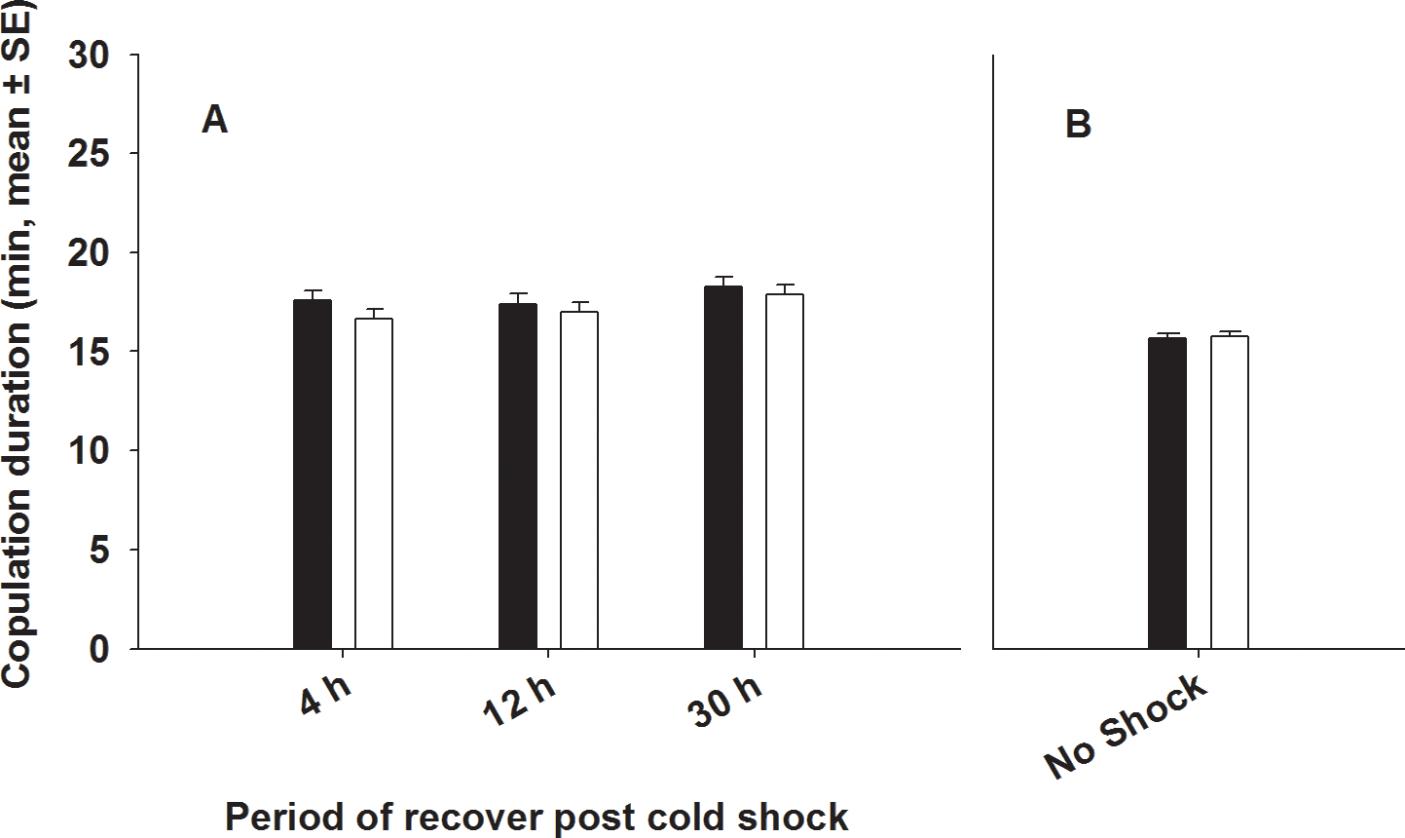
**Effect of cold shock (A) or no shock (B) on copulation duration**. (A) We assayed copulation duration 4 h, 12 h and 30 h post cold shock. Closed bars represent FCB and open bars represent FSB populations. Selection, period and Selection × period had no significant effect on copulation duration. (B) Under no-shock treatment, there was no significant difference in copulation duration between FSB and FCB males.

Copulation duration was unresponsive to selection. None of the main effects or the interactions terms were significant (Table 1B, Fig 2A).

#### (c) Mating success

**Fig 3:**
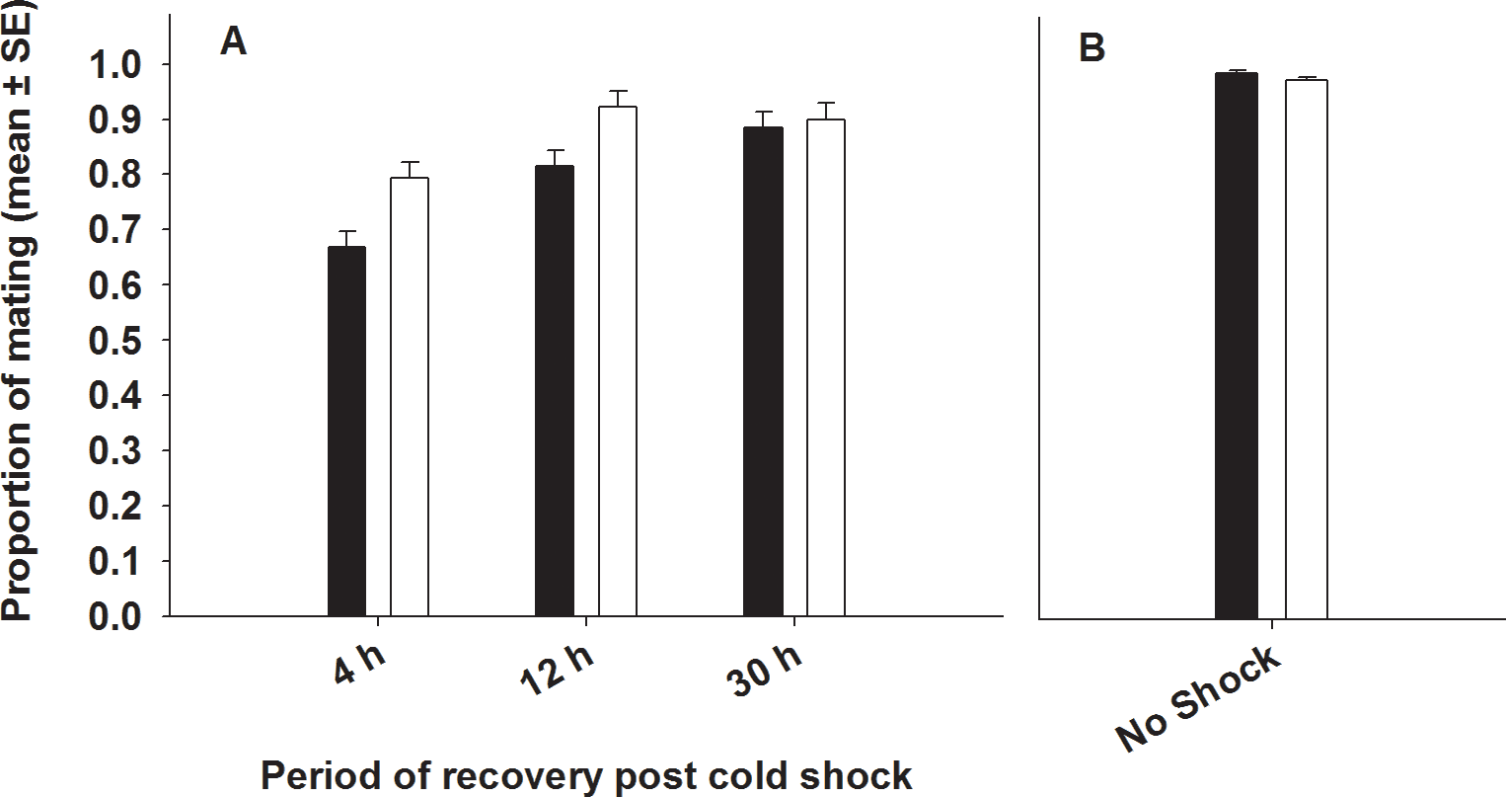
**Effect of cold shock (A) or no shock (B) on mating success**. Closed bars represent FCB and open bars represent FSB populations. (A) Selection and period had significant effects on mating success. However, Selection × period interaction was not significant. (B) Under no-shock treatment, there was no significant difference in mating success between FSB and FCB males.

We found significant effects of selection and period on mating success (Table 2A, Fig 3A). Post cold shock, significantly more (~8%) FSB males mated successfully with base line females relative to FCB males (Fig 3A). Mating success increased with increase in the time of recovery post cold shock. Multiple comparisons using Tukey’s HSD suggested that both FSB and FCB males subjected to cold shock had lower mating success (~9% and ~12%) after 4 hours of recovery relative to 12 hours and 30 hours of recovery. However, none of the interactions were significant (Table 2A, Fig 3A).

**Table 2:** Effect of cold shock on pre- and post-copulatory traits. Summary of results of a three-factor mixed model ANOVA considering selection regime (FSB and FCB) and period (recovery period 4, 12 and 30 hours after cold shock) as fixed factors crossed with block as random factor on the (A) mating success and (B) male fertility data. *p*-values in bold are statistically significant. Estimated denominator DF (Satterthwaite method) was very low. Hence *F* ratio and *p* values are unavailable for some of the effects.

Trait	Effect	SS	MS Num	DF Num	DF Den	*F* ratio	*p*
(A)	Selection (Sel)	0.051	0.051	1	4	17.154	**0.014**
Mating	Period (Per)	0.152	0.076	2	8	18.937	**0.001**
success	Block (Blk)	0.078	0.020	4	1.127	7.214	0.243
	Sel×Per	0.017	0.008	2	8	1.986	0.199
	Sel×Blk	0.012	0.003	4	8	0.696	0.616
	Per×Blk	0.032	0.004	8	8	0.937	0.535
	Sel× Per×Blk	0.034	0.004	8	.	.	.
(B)	Selection (Sel)	0.053	0.053	1	4	66.681	**0.001**
Male	Period (Per)	0.052	0.026	2	8	8.104	**0.012**
fertility	Block (Blk)	0.016	0.004	4	0.588	.	.
	Sel×Per	0.020	0.010	2	8	1.694	0.244
	Sel×Blk	0.003	0.001	4	8	0.136	0.965
	Per×Blk	0.026	0.003	8	8	0.551	0.792
	Sel×Per×Blk	0.047	0.006	8	.	.	.

#### (d) Male fertility

**Fig 4:**
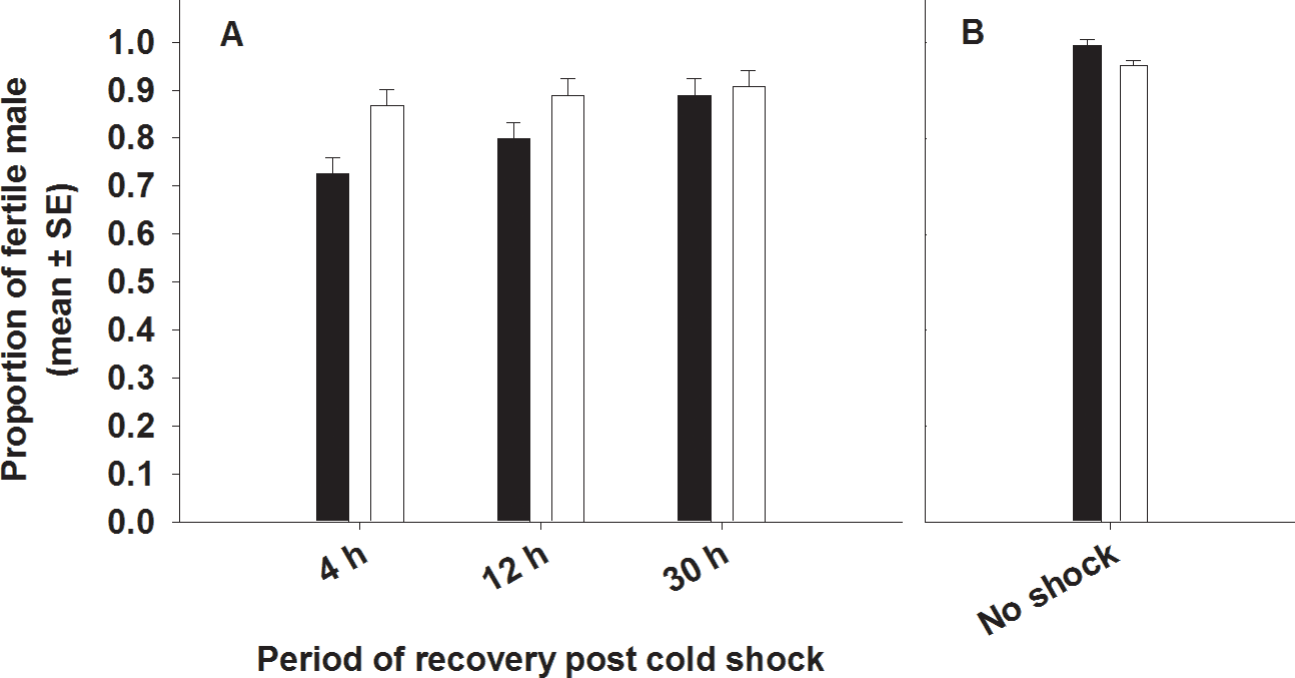
**Effect of cold shock (A) or no shock (B) on male fertility**. Closed bars represent FCB and open bars represent FSB populations. If a female mated to a given male produced at least one egg that hatched, the male was considered to be fertile. (A) Selection and period had significant effect on male fertility. However, selection × period interaction was not significant. (B) Under no-shock treatment, there was no significant difference in male fertility between FSB and FCB males.

We found a significant effect of selection and period on male fertility. FSB males were significantly more fertile (~8.5%) than FCB males (Table 2B, Fig 4A). Multiple comparisons employing Tukey’s HSD indicated that male fertility increased with increase in the time of recovery post cold shock (Fig 4A). When females were mated to males (FSB and FCB) that had recovered for different periods from cold shock, we found that fewer females mated to the males that had recovered for four hours post cold shock laid fertile eggs compared to females mated to males that had recovered for 12 or 30 hours. However, none of interactions were significant.

### Experiment 1.2: Progeny production of ancestral females mated with cold shocked males

**Fig 5:**
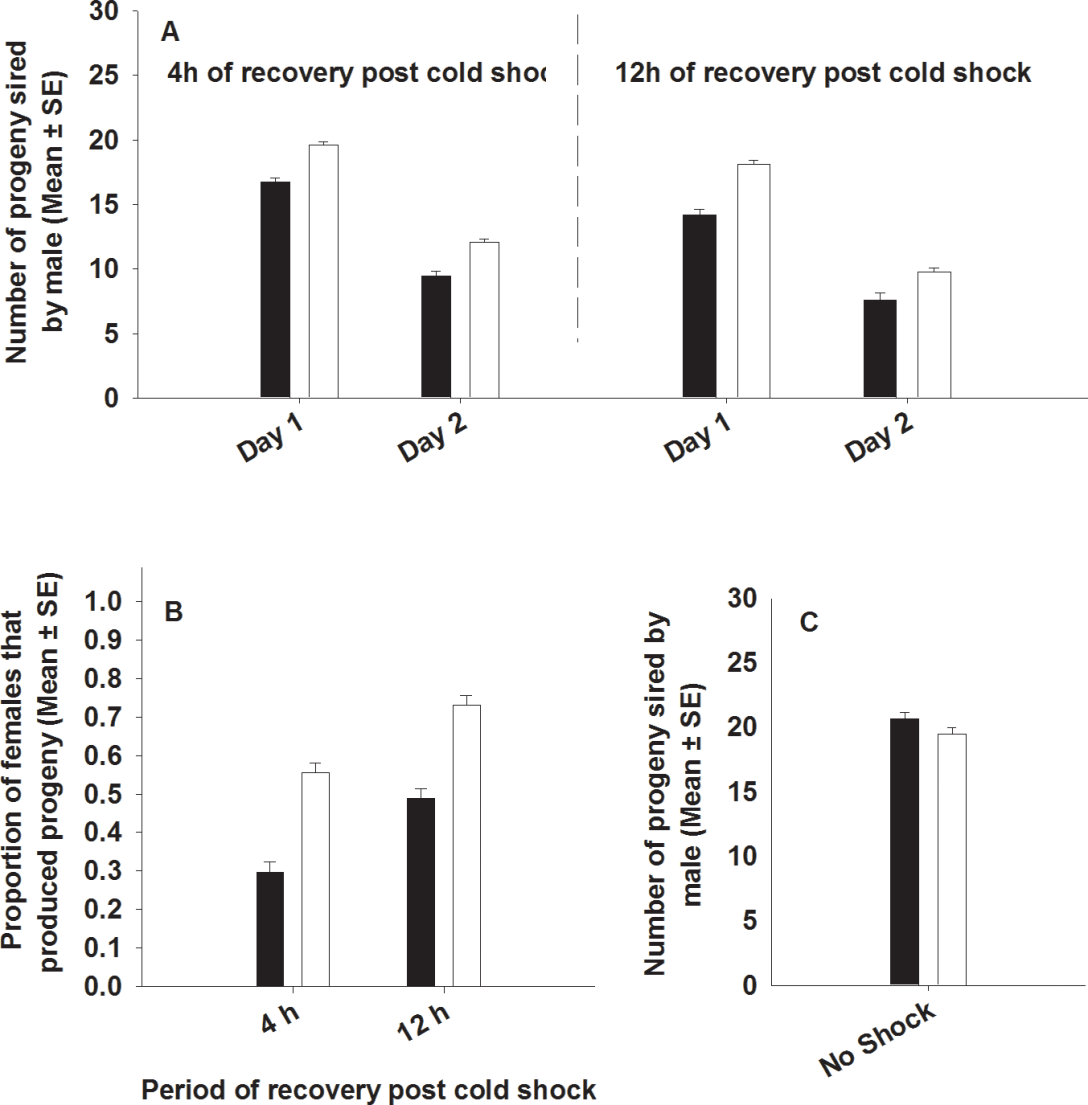
**Effect of cold shock (A and B) or no shock (C) on progeny production**. Closed bars represent FCB and open bars represent FSB populations. (A) Number of progeny produced by (ancestral) BRB females exposed to FSB or FCB males subjected to cold shock and allowed to recover for 4 or 12 hours. Selection and day had significant effect on the progeny production. (B) The proportion of ancestral females which had produced progeny after being exposed to FSB or FCB males that were cold shocked and allowed to recover for 4 or 12 hours. Selection and period had significant effect on the proportion of females that produce zero progeny after being exposed to cold shocked male. However, two way interaction of selection × period was not significant. (C) Under no shock treatment, there was no significant difference in the number of progeny sired by FSB and FCB males when exposed to ancestral BRB females.

The number of progeny produced by females was measured separately for two days (day one fitness and day two fitness). We found that selection and period had a significant effect on female progeny production (Table 3, Fig 5A). Females mated with FSB population males had a significantly higher progeny production compared to females mated with FCB population males. Period had a significant effect on female progeny production. Progeny production increased with the time of recovery. Ancestral females mated to males that had recovered for 4 hours following cold shock had significantly lower progeny production comparative to females mated to males that had recovered for 12 hours post cold shock. Females produced more progeny on day one compared to day two, but the difference was not significant. None of the interactions were significant (Table 3, Fig 5A).

**Table 3:** Progeny production of ancestral females after exposed with cold shocked males. Summary of results from a four-factor ANOVA using selection regime (FSB and FCB), period (recovery period 4 hours and 12 hours post cold shock) and day (progeny production on day1 and day 2) as fixed factors crossed with block (1–5) as random factor on the progeny production of females mated to FSB or FCB males (number of progeny from each vial was used as unit of analysis). *p*-values in bold are statistically significant.

Effect	SS	MS Num	DF Num	DF Den	*F* ratio	*p*	
Selection (Sel)	1964.166	1964.166	1	4.125	8.251		**0.044**
Day	13025.704	13025.704	1	4.526	215.367		**<0.001**
Block (Blk)	295.721	73.930	4	4.727	0.196		0.930
Period (Per)	987.046	987.046	1	4.148	4.913		0.089
Sel×day	62.607	62.607	1	4.571	1.114		0.344
Sel×Blk	1005.593	251.398	4	5.591	3.051		0.115
Sel×Per	7.164	7.164	1	4.839	0.179		0.690
Day×Blk	243.893	60.973	4	6.211	0.568		0.696
Day×Per	0.181	0.181	1	4.501	0.003		0.960
Blk×Per	846.199	211.550	4	5.613	2.351		0.174
Sel×Day×Blk	225.466	56.367	4	4	4.375		0.091
Sel×Day×Per	35.311	35.311	1	6.777	2.258		0.178
Sel×Blk×Per	155.685	38.921	4	4	3.021		0.155
Day×Blk×Per	255.795	63.949	4	4	4.964		0.075
Sel×Day×Blk×Per	51.531	12.883	4	1155	0.240		0.916

We calculated the proportion of females that produced progeny after being exposed to FSB or FCB males post cold shock. We found significant effects of selection and period on the proportion of females that produced progeny. About 65% of the females produced progeny after being exposed to cold shocked FSB males, whereas ~40% of the females produced progeny after being exposed to cold shocked FCB males. Period had significant effect. Approximately 43% females exposed to FSB or FCB males that had recovered for 4 hours post cold shock produced progeny, whereas 61% of females exposed to FSB or FCB males that had recovered for 12 hours post cold shock produced progeny. None of the interactions were significant (Table 4, Fig 5B).

**Table 4:** Proportion of ancestral females that produced progeny after exposing with cold shocked males. Summary of results from a three-factor ANOVA on proportion of females that produced progeny (post mating with FSB or FCB males) using selection regime (FSB and FCB), period (recovery period 4 hours and 12 hours post cold shock) as fixed factors crossed with block (1–5) as random factor. *p*-values in bold are statistically significant.

Effect	SS	MS Num	DF Num	DF Den	*F* ratio	*p*
Selction (Sel)	0.313	0.313	1	4	18.654	**0.012**
Block (Blk)	0.114	0.029	4	5.068	1.353	0.366
Period (Per)	0.168	0.168	1	4	21.964	**0.009**
Sel×Blk	0.067	0.017	4	4	5.047	0.073
Sel×Per	3×10^−4^	3×10^−4^	1	4	0.101	0.766
Blk×Per	0.031	0.008	4	4	2.294	0.221
Sel×Blk×Per	0.013	0.003	4	.	.	.

### Experiment 1.3: Pre- and post-copulatory traits in males not subjected to cold shock

We assayed- (A) Mating latency, (B) Copulation duration, (C) Mating success, (D) Male fertility and (E) Male effect on progeny production by (ancestral) females mated to FSB and FCB males not subjected to cold shock. We found that the FSB and FCB males did not differ significantly in any of these traits, indicating that there are no differences in the basal levels of these traits between the males of the two populations (FSB and FCB) (Table 5A–5E, Figs 1B, 2B, 3B, 4B and 5C)

**Table 5:** Pre- and post-copulatory traits in males not subjected to cold shock. Summary of the results from a two-factor mixed model ANOVA on (A) mating latency (B), copulation duration (C) mating success, (D) male fertility and (E) progeny production. Data considering selection regime (FSB and FCB) as the fixed factors crossed with block (1–5) as random factor. *p*-values in bold are statistically significant.

Trait	Effect	SS	MS Num	DF Num	DF Den	*F ratio*	*p*
(A)	Selection (Sel)	1.207	1.207	1	4.005	0.070	0.804
Mating	Block (Blk)	870.409	217.602	4	4	12.617	**0.015**
latency	Sel×Blk	68.988	17.247	4	322	1.102	0.356
(B)	Selection (Sel)	0.605	0.605	1	4.005	0.066	0.811
Copulation	Block (Blk)	154.364	38.591	4	4	4.182	0.097
duration	Sel×Blk	36.911	9.228	4	322	1.249	0.290
(C)	Selection (Sel)	3.3×10^−4^	3.3×10^−4^	1	4	2.268	0.207
Mating	Block (Blk)	1.4×10^−3^	3.4×10^−4^	4	4	2.385	0.210
success	Sel×Blk	5.7×10^−4^	1.4×10^−4^	4	.	.	.
(D)	Selection (Sel)	4.6×10^−3^	4.6×10^−3^	1	4	7.064	0.057
Male	Block (Blk)	3.1×10^−3^	7.7×10^−4^	4	4	1.189	0.435
fertility	Sel×Blk	2.6×10^−3^	6.5×10^−4^	4	.	.	.
(E)	Selection (Sel)	120.915	120.915	1	4.006	2.915	0.163
Progeny	Block (Blk)	8160.108	2040.027	4	4	49.195	**0.001**
production	Sel×Blk	165.873	41.468	4	325	0.692	0.598

### Experiment 2: Effect of selection regime on Sperm offense (P2) ability

**Fig 6:**
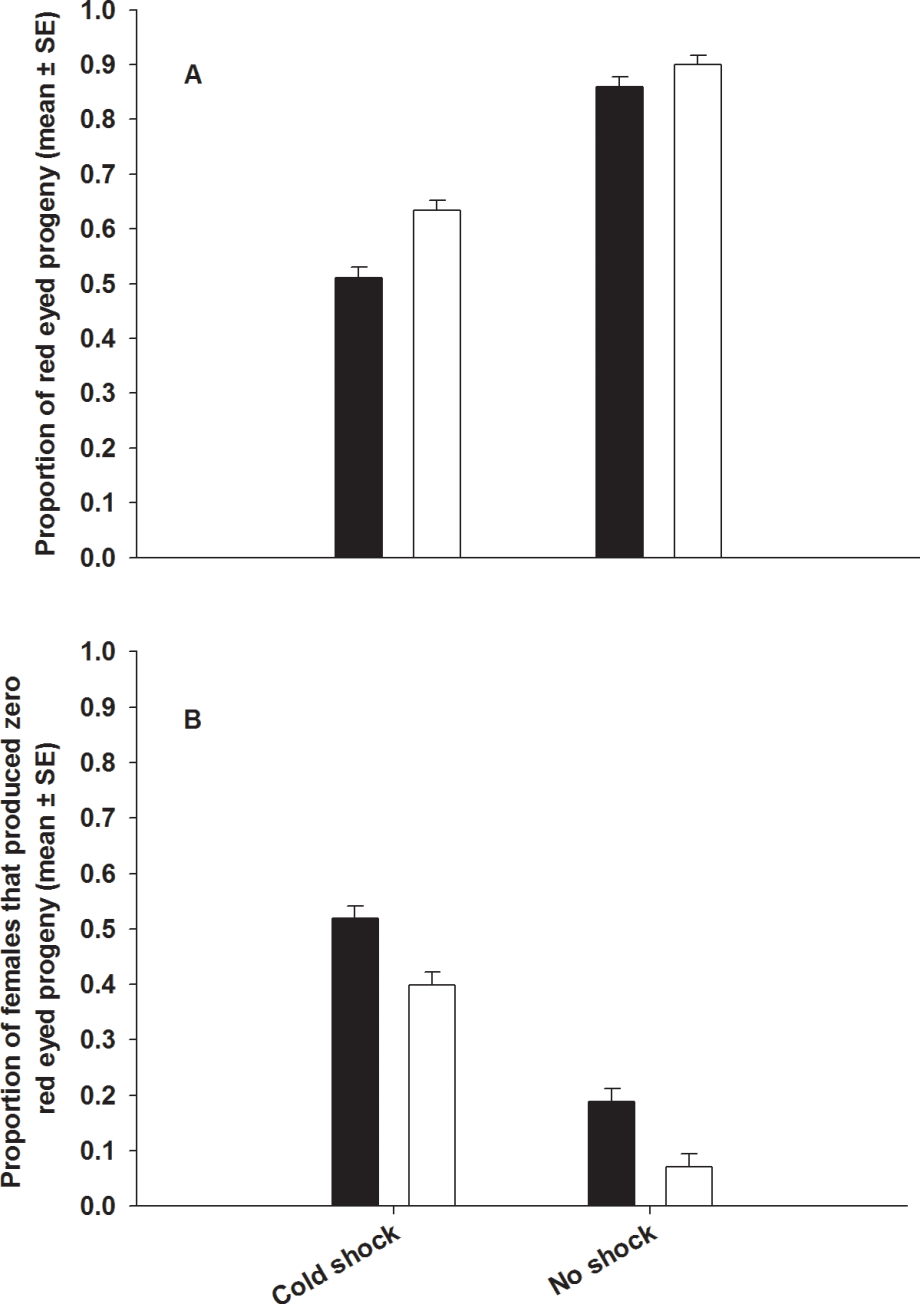
Effect of cold shock on sperm offense ability (P2). Closed bars represent the FCB and open bars represent the FSB populations. Since the females and competitors males have recessive scarlet eye marker and the FSB and FCB males have dominant red eye marker, progeny sired by FSB and FCB males will show red eye color. Hence, in this experiment, the proportion of red eyed progeny is an indicator of sperm offense ability. (A) These data come from females that produced at least one progeny from the FSB or FCB male (that is non-zero sperm offense ability). Compared to FCB males, FSB males had higher sperm offense ability under cold shocked and non-shocked conditions. Selection and treatment had significant effect on P2. However, selection × treatment interaction was not significant. (B) Proportion of males that had zero-sperm offense ability (P2). Significantly lesser proportion of FSB males had zero sperm offense compared to FCB males. Selection and treatment effects were significant. However, selection × treatment interaction was not significant.

We found a significant effect of selection and treatment on sperm offense ability. Post cold shock, FSB males sired 12% more progeny compared to FCB males (Fig 6A). Without cold shock, FSB males sired 4% more progeny relative to FCB males, although this difference was not significant (Fig 6A). In both FSB and FCB populations cold shocked males had lower sperm offense ability (P2) compared to non shocked males. None of interactions were significant (Table 6A).

**Table 6:** Effect of cold shock on sperm offense ability. Summary of results from a three-factor mixed model ANOVA treating selection regime (FSB and FCB) and treatment (cold shock and no shock) as fixed factors crossed with block (1–5) as a random factor on (A) sperm offense ability and (B) proportion of females which produced zero red eyed progeny. *p*-values in bold are statistically significant.

Traits	Effect	SS	MS Num	DF Num	DF Den	*F* ratio	*p*
(A)	Selection (Sel)	1.254	1.254	1	4.02	21.538	**0.01**
Offense	Block (Blk)	2.602	0.650	4	2.548	5.451	0.121
ability	Treatment (Trt)	17.523	17.523	1	4.009	142.599	**<0.001**
	Sel×Blk	0.233	0.058	4	4	0.94	0.523
	Sel×Trt	0.316	0.316	1	4.018	5.099	0.087
	Blk×Trt	0.492	0.123	4	4	1.987	0.261
	Sel×Blk×Trt	0.248	0.062	4	737	0.908	0.459
(B)	Selection (Sel)	0.070	0.070	1	4	9.211	**0.039**
Proportion of	Block (Blk)	0.014	0.004	4	2.809	0.511	0.739
females that	Treatment (Trt)	0.540	0.540	1	4	266.796	**<0.001**
produced	Sel×Blk	0.031	0.008	4	4	2.861	0.167
zero red eyed	Sel×Trt	1×10^−5^	1×10^−5^	1	4	0.004	0.954
progeny	Blk×Trt	0.008	0.002	4	4	0.757	0.603
	Sel×Blk×Trt	0.011	0.003	4	.	.	.

A greater proportion of the FCB males had sired no progeny under competitive conditions compared to FSB males. This is clear from the fact that a greater proportion of females mated to FCB males produced no red eyed progeny compared to females mated to FSB males. This trend prevailed regardless of whether the males were subjected to cold shock or not (Table 6B, Fig 6B).

## Discussion

In the present study, we have assessed the evolution of pre and post-copulatory traits of males in populations of *D*. *melanogaster* selected for cold shock resistance. Our results clearly indicate that post cold shock, FSB males (when exposed to ancestral females) took less time to start mating, had a higher mating success, were more fertile and produced more progeny relative to FCB males. Post cold shock, FSB males also had higher sperm competitive ability when compared to FCB males. However, unlike other traits, copulation duration was not different between FSB and FCB males. When the males were not subjected to cold shock, there was no difference in mating latency, copulation duration, mating success, or progeny production between FSB and FCB males.

Mating latency and mating success are affected both by the ability of the male to induce the female to mate as well as the female’s eagerness to mate. In the present study, FSB and FCB males were provided with common, non-cold shocked, ancestral females. Hence, differences in mating latency and mating success would represent inherent differences in the FSB and FCB males’ ability to successfully mate and/or females’ mating preference across these two types of males. Both high and low temperature treatments are known to affect mating latency [34]. Both heat stress and cold stress are known to reduce male mating success [31, 36]. In agreement with these results, we found that cold shocked males (both FSB and FCB) show higher mating latency and lower mating success relative to males not subjected to cold shock. As males were allowed to recover from the cold shock, mating latency decreased while mating success increased. However, post cold shock, FSB males took less time to start mating (lower mating latency) and were more successful at mating (higher mating success) compared to FCB males. Given that there were no differences in mating latency and mating success of FCB and FSB males under no shock conditions, there are two possible explanations (not mutually exclusive) for the observed results; (a) FSB males recover from cold-shock at a faster rate compared to FCB males. This is consistent with the observation that the FCB males move closer to the FSB males in terms of their mating latency and mating success values with increasing durations of recovery. (b) FSB males are better protected against injury from cold shock and hence suffer lesser damage due to cold shock. Populations of *D*. *melanogaster* selected for resistance to cold shock are known to have evolved increased levels of specific metabolites such as glycogen, trehalose and proline which are known to act as cryoprotectants [41]. Similarly, it is possible that FSB populations have evolved mechanisms to protect themselves against cold shock induced damage. It is important to note that these possibilities are not mutually exclusive.

In many insects, including *D*. *melanogaster*, selection for resistance to certain kinds of stress leads to increase in body size [42, 43]. At least some studies show that in *Drosophila*, larger males have better mating success [44–50]. Thus, selection for increased stress resistance can increase mating success through its effects on body size. This, however, is unlikely to be an explanation in our study since we find no difference between the body size of the FSB and FCB populations (details are provided in Table A and Figure A in S1 File). Mating in *Drosophila* requires complex courtship behaviours to be executed by the males while cold shock impairs locomotor ability of flies. Therefore, populations that can recover faster (in terms of mobility) have a greater chance of successfully courting and mating. Previous studies have documented the evolution of faster rate of recovery from chill coma in terms of mobility in the populations of *D*. *melanogaster* selected for increased resistance cold stress or heat stress [51–53]. Similarly, it is quite possible that the FSB populations have evolved greater ability to regain mobility post cold shock and can hence mate early.

While copulation is necessary, it is not sufficient to ensure the fitness of a male. The male should be able to successfully transfer functional ejaculate during copulation to the females. Copulation duration is often used as a measure of the amount of ejaculate transferred during copulation. It is known to be positively correlated with the amount of some components of ejaculate transferred during copulation [54]. We found no difference between FSB and FCB males in copulation duration indicating that the amount of ejaculate transferred was probably not different. We further analysed the effectiveness of the males in transferring a functional ejaculate by assessing two traits—male fertility and progeny production.

Cold shock reduces male fertility by killing/immobilising the sperm or affecting the sperm quality [36]. When young *Drosophila* males are subjected to cold shock, progeny production is reduced, compared to males not subjected to cold shock [55]. Our results also show that cold shock reduces male fertility and that the females mated to males subjected to cold shock produce less number of progeny. However, we find that post cold-shock, male fertility and female progeny production are higher in FSB males relative to FCB males. It is important to note that under the ‘no-shock’ treatment, there are no significant differences between FSB and FCB males in their fertility or progeny production. Male fertility and progeny production require that functional sperm are transferred to the females during copulation. Previous studies indicate that when males are subjected to a cold shock of -5°C for one hour, no motile sperm are found in their reproductive tracts for the next 24 hours [36]. In our study, at least some functional sperm are transferred by males (FSB and FCB) during copulation even within 4 hours post cold shock. The higher fertility and progeny production in FSB males post cold shock could be because (a) FSB males are better at protecting sperm from cold- induced damage (b) FSB males produce more sperm but are not necessarily better at protecting the sperm from cold-induced damage (c) FSB males can produce functional sperm at a faster rate post cold shock. Given that cold shocked males produce progeny even within 4 hours post cold shock and that sperm production is generally expected to be a lengthy process, it is unlikely that option (c) alone would account for the observed differences.

Post cold shock, FSB males show higher sperm offense ability compared to FCB males. This is consistent with the idea that the FSB populations probably produce more sperm and/or are better at protecting sperm from cold damage. This result is also in agreement with our own previous studies where we found that post cold shock, FSB males mated with a higher number of non-virgin females and sired more progeny compared to FCB males [37]. An un-explored aspect of the effect of cold shock on sperm competitive ability is the effect of cold shock on accessory gland proteins. Seminal plasma proteins are known to protect sperm from cold induced damage in some mammalian species [56] (but also see [57, 58]). If insect ejaculate proteins have a similar cryoprotective function, the faster recovery of FSB males in terms of mating traits could also be due to the evolution ejaculate proteins. It is to be emphasised here that the differences in mating rate post cold shock are enough to explain the differences in sperm offense ability (as measured in our study). Specifically, if FSB males mate more often with females post cold shock (compared to FCB males), then, given the strong last male sperm precedence in this species, FSB would have higher sperm offense ability compared to FCB males, even without any changes in sperm number, physiology or Acps. Since in this assay we did not observe the number times the flies mated with the second male, we cannot account for this possibility.

## Conclusions

Our study shows that in absence of cold shock, males from the FSB and FCB populations have identical reproductive behavior and fitness, but upon cold shock, FSB males are better at reproductive recovery in terms of mating latency, mating success, male fertility, progeny production and sperm offense ability. These findings indicate adaptive evolution of the reproductive traits in males in response to selection for resistance to cold shock. Central results of this study help us in understanding the evolution of reproductive traits in response to environmental stresses.

## Supporting Information

S1 File Effect of selection on male dry body weight. (DOCX)
