# Supplementary material for: Evolution of Pre- and Post-Copulatory Traits in Male Drosophila melanogaster as a Correlated Response to Selection for Resistance to Cold Stress
Source: PLoS One. 2016 Apr 19;11(4):e0153629. doi: 10.1371/journal.pone.0153629 (PMC4836659; doi:10.1371/journal.pone.0153629)
Supplement: S1 File — (DOCX) [file pone.0153629.s001.docx]

**S1 File: Effect of selection on male dry body weight.**

**Experiment 1: Dry body weight measurement**

Dry body weight was assayed after 33 generations of selection. Experimental flies were generated following two generations of standardization. Standardized flies were provided with a fresh banana-yeast-jaggery food plate for one hour to allow females to lay stored eggs. A fresh plate was again provided for four hours. Eggs laid on the second plate were used to generate experimental flies. The second set of plates containing eggs were then incubated at standard conditions (25^o^C temperature, 50-60% relative humidity, and 12 hours-12 hours light/dark cycle) for 18 hours to allow them to hatch into first instar larvae. The larvae were collected (using a moist brush) into vials with 6 ml banana-yeast-jaggery food. For each population,10 replicate vials at a density of 30 larvae per vial were established. The vials were incubated at standard laboratory conditions. The vial positions were randomized and moved daily within the incubator. Once pupae formed, each vial was manually scanned every 2 hours. Freshly eclosed flies were flash frozen using liquid nitrogen and stored at -80^o^C until dry body weight measurement. Five flies of a given sex were grouped together, dried in a hot air oven at 65^o^C for 48 hours and weighed in a high precision electronic balance (Sartorius CPA225D). For each population, ten such sets were weighed. Thus, a total of 50 males per population were used for measurement of dry body weight. Body weight of each group of five flies was used as the unit of analysis.

**Data analysis:**

Dry body weight of males was analyzed using a two-factor mixed model ANOVA treating selection regime (FSB vs. FCB) as fixed factor crossed with random block (1-5). All the analyses were done using JMP 10 (SAS Institute, Cary, NC, USA).

**Results**

Males mean dry weight analysis revealed that there was no significant main effect of selection. This suggests that body size at eclosion has not evolved in our populations in response to selection for resistance against cold stress.

**Table A: Effect of selection on male dry body weight.**

Summary of results from a two-factor mixed model ANOVA on the mean dry body weight of males using Selection (FCB and FSB) as fixed factor crossed with random Block (1-5). *p*-values in bold are statistically significant. SS: Numerator sum of squares, MS Num: Numerator mean square, DF Num: Numerator degrees of freedom, DF Den: Denominator degrees of freedom.

| **Effect** | **SS** | **MS Num** | **DF Num** | **DF Den** | **F *ratio*** | ***p*** |
| --- | --- | --- | --- | --- | --- | --- |
| Selection (Sel) | 1.6 ×10^-5^ | 1.6 ×10^-5^ | 1 | 4.000 | 0.122605 | 0.744 |
| Block (Blk) | 8.3×10^-3^ | 2.0×10^-3^ | 4 | 4.000 | 15.89931 | **0.010** |
| Sel × Blk | 5.2×10^-4^ | 1.310^-4^ | 4 | 90.000 | 0.482825 | 0.749 |

**Figure A. Effect of selection on male dry body weight.**

Dry body weight at eclosion of males from the FSB and FCB populations. Selection did not have significant effect on mean dry body weight of males.
